# Beyond direct exposure to violence: effects of living in disordered and violent communities on psychological distress in young Mexican people

**DOI:** 10.1590/0102-311XEN058123

**Published:** 2024-02-02

**Authors:** Miguel A. Pérez-Sastré, Carmen García-Peña, Luciana Ramos-Lira, Luis Ortiz-Hernández

**Affiliations:** 1 Doctorado en Ciencias Médicas, Odontológicas y de Salud, Universidad Nacional Autónoma de México, Ciudad de México, México.; 2 Instituto Nacional de Geriatría, Ciudad de México, México.; 3 Instituto Nacional de Psiquiatría Ramón de la Fuente Muñiz, Ciudad de México, México.; 4 Universidad Autónoma Metropolitana - Unidad Xochimilco, Ciudad de México, México.

**Keywords:** Violence, Environment, Psychological Distress, Mental Health, Youth, Violencia, Ambiente, Distrés Psicológico, Salud Mental, Juventud, Violência, Meio Ambiente, Angústia Psicológica, Saúde Mental, Juventude

## Abstract

The association between community violence and mental health has been studied by reports of individual experiences, particularly in adolescents and youths, but little is known about the effect of living in disordered and violent communities. This study aims to determine the possible relation between living in disordered and violent community environments and psychological distress in Mexican adolescents and youths regardless of their individual experience of victimization and to assess the potential modifying effect of sex and age on this association. Data come from a cross-sectional survey with a representative sample of adolescents and youths living in Mexican municipalities, including 39,639 participants aged from 12 to 29 years. Disordered and violent community environments were assessed using reports from a secondary sample of adults who lived in the same communities as participants. Using exploratory factor analysis, three contextual variables related to disordered and violent community environment were created: social disorder, vandalism, and criminality. Multilevel linear regression models with random intercept were estimated. Adolescents and youths who lived in environments with higher social disorder had more psychological distress. Men in environments with greater vandalism had a higher level of psychological distress. Unexpectedly, women from communities with higher levels of crime had fewer symptoms. It is necessary to address the violence that exists in these communities, creating strategies that reduce not only crime, but also the social disorder and vandalism that could contribute to developing negative effects on mental health.

## Resumen

La asociación entre la violencia comunitaria y la salud mental se ha evaluado mediante informes de experiencias individuales, especialmente de adolescentes y jóvenes, pero poco se sabe sobre el efecto de residir en comunidades desordenadas y violentas. El objetivo de este estudio fue comprobar si existe una relación entre residir en entornos comunitarios desordenados y violentos y el distrés psicológico en adolescentes y jóvenes mexicanos, independientemente de su experiencia individual de victimización, así como evaluar el posible efecto modificador del sexo y la edad en esta asociación. Los datos provienen de una encuesta transversal que tomó como muestra representativa a 39.639 adolescentes y jóvenes de entre 12 y 29 años, residentes en ciudades mexicanas. Los entornos comunitarios desordenados y violentos se evaluaron mediante informes de una muestra secundaria de adultos que residían en las mismas comunidades donde vivían los participantes. El análisis exploratorio de datos posibilitó crear tres variables contextuales relacionadas con el entorno comunitario desordenado y violento: desorden social, vandalismo y delincuencia. Se estimaron modelos de regresión lineal multinivel con intercepto aleatorio. Los adolescentes y jóvenes que residían en ambientes con mayor desorden social presentaron mayor distrés psicológico. Los varones en entornos con más vandalismo tenían un mayor nivel de distrés psicológico. Inesperadamente, las mujeres que viven en comunidades con mayores niveles de delincuencia tuvieron menos síntomas. Es necesario enfrentar la violencia existente en las comunidades para generar estrategias que reduzcan no solo la delincuencia, sino también el desorden social y el vandalismo que pueden contribuir al desarrollo de efectos negativos en la salud mental.

## Resumo

A associação entre violência comunitária e saúde mental tem sido estudada por meio de relatos de experiências individuais, particularmente em adolescentes e jovens, mas pouco se sabe sobre o efeito de viver em comunidades desordenadas e violentas. O objetivo deste estudo é determinar se há relação entre viver em ambientes comunitários desordenados e violentos e estresse psicológico em adolescentes e jovens mexicanos, independentemente de sua experiência individual de vitimização, e avaliar o potencial efeito modificador do sexo e da idade sobre essa associação. Os dados são de uma pesquisa transversal com uma amostra representativa de adolescentes e jovens residentes em cidades mexicanas, incluindo 39.639 participantes com idades de 12 a 29 anos. Ambientes comunitários desordenados e violentos foram avaliados por meio de relatos de uma amostra secundária de adultos que viviam nas mesmas comunidades onde os participantes viviam. Por meio da análise exploratória de dados, foram criadas três variáveis contextuais relacionadas ao ambiente comunitário desordenado e violento: desordem social, vandalismo e criminalidade. Foram estimados modelos de regressão linear multinível com interceptação aleatória. Adolescentes e jovens que viviam em ambientes com maior desordem social apresentaram maior estresse psicológico. Homens em ambientes com mais vandalismo apresentaram maior nível de estresse psicológico. Inesperadamente, as mulheres de comunidades com níveis mais altos de criminalidade tiveram menos sintomas. É preciso enfrentar a violência existente nas comunidades, gerando estratégias que reduzam não só a criminalidade, mas também a desordem social e o vandalismo que possam contribuir para o desenvolvimento de efeitos negativos na saúde mental.

## Introduction

Mental health disorders have become of interest to public health due to their increasing frequency ^1^. Mexico holds a high prevalence of mental disorders. The lifetime prevalence of depression in the population aged 12 years and older is 32.5% ^2^. At the same time, the rate of community violence has remained high, with crime incidence rates exceeding 35,000 crimes per 100,000 inhabitants/year from 2012 to 2017 ^3^. These figures are an underestimation of the problem due to the high percentage of unreported crimes, estimated at around 93% in the last five years ^4^. Adolescents and youths are more exposed to community violence than adults ^5^ ^,^ ^6^ ^,^ ^7^. Moreover, they have a high prevalence of mental disorders such as depression, anxiety, substance use, and suicide in comparison to general population ^1^.

Community violence is any deliberate act (including threats and use of force) to cause physical harm to a person or persons in a community or neighborhood ^5^ ^,^ ^8^ ^,^ ^9^. It can be experienced directly (victimization) or indirectly (witnesses and hearing about). Other acts imply that informal rules of order are being broken, decreasing the sense of respect and civility ^10^ ^,^ ^11^. These incivilities people experience include physical, such as vandalism, and social ones, such as disruptive disputes, careless neighbors, loitering, and alcohol consumption, which may increase exposure to community violence ^12^.

Individuals who have experienced or witnessed community violence are more likely to experience mental health-related problems ^13^ ^,^ ^14^. Also, people who are aware of the acts of community violence in their neighborhood could suffer the effects on their mental health, even without being victims or witnesses ^15^ ^,^ ^16^. In other words, it must be recognized that community violence not only affects people who suffer from it directly, but that it also creates a social environment that affects all the inhabitants of a locality. Interpersonal violence, vandalism, and social disorder create a social environment that is adverse to the security feeling required to conduct daily activities peacefully.

People who are aware of community violence in their neighborhoods and perceive themselves as vulnerable could develop neurotransmitter hyperactivity. This state produces a sustained alert, placing the individual in a hypersensitivity or aggression state accompanied by anxiety, which will favor the development of psychological distress ^17^. Psychological distress can encourage social isolation, contributing to the development of mental disorders ^17^ ^,^ ^18^. Consequently, it is possible to assume that the negative effect of community violence in mental health could stem from different mechanisms.

Evidence shows that, as expected, the higher the exposure to community violence, the greater the negative mental health outcomes ^19^. However, most studies that evaluated this relation tends to define exposure to community violence as victimization or the witnessing of violent acts in a community. Few studies have considered the effect of specifically living in a violent community on mental health over and above individual victimization experiences but results are inconsistent ^15^ ^,^ ^16^ ^,^ ^20^ ^,^ ^21^ ^,^ ^22^ ^,^ ^23^ ^,^ ^24^ ^,^ ^25^. Contextual variables must be employed to know this effect. Most of these studies used criminal or homicide records or indexes based on these records to confirm this type of variables and assess their relation with mental health ^15^ ^,^ ^16^ ^,^ ^20^ ^,^ ^23^. Overall, three studies found the expected relationship between living in a violent community and negative mental health outcomes ^15^ ^,^ ^16^ ^,^ ^20^. In Scotland, higher exposure to crime was associated with a higher likelihood of reporting mental disorder and the prescription of antipsychotic drugs ^16^. Colombian adolescents who lived in places with more homicides showed more mental disorders ^15^. Higher crime rates were associated with higher depression symptoms in American adolescents ^20^. In a study carried out in the United States population, violent crime was not associated with internalizing symptoms such as depression or anxiety ^23^.

Moreover, two other studies in American people (one with adolescents ^24^ and one with youths ^22^), measured exposure to a violent community by parental reports. The study with youths found that higher exposition to community violence was associated with more depression and anxiety symptoms ^22^, whereas the study with adolescents found no relation between community violence and internalizing symptoms ^24^. Finally, only one study used a community sample composed of adults from the same places as participants, finding the expected relationship, i.e., exposure to community violence was related to more internalizing symptoms ^21^.

Age and sex could modify the relation between living in a violent community and psychological distress. Women are more likely to have negative mental health outcomes than men ^1^ ^,^ ^26^, but the latter are more exposed to community violence ^27^. Similarly, youths have a higher exposure to community violence than adolescents ^5^ ^,^ ^28^, although they also have better coping mechanisms ^17^.

Therefore, the analysis of the relation of living in a disordered and violent community environments over and above individual victimization experiences with psychological distress will give us a comprehensive overview to recognize if effects only stem from being a victim of violence or also from inhabiting community violence environments.

This study aimed to determine the possible relationship between living in a disordered and violent community environment measured at a contextual level and the presence of psychological distress in Mexican adolescents and youths regardless of their experiences of interpersonal community violence. We also sought to evaluate the potential modifying effect of sex and age in this relationship.

## Material and methods

### Study design

A secondary analysis of the *Survey of Social Cohesion for the Prevention of Violence and Crime* (ECOPRED, acronym in Spanish) databases was developed. ECOPRED is a cross-sectional survey carried out by the Mexican National Institute of Geography and Statistics (INEGI, acronym in Spanish) that targeted young Mexicans living in large cities. The data were collected from October 6 to December 9, 2014, and included households from 47 cities across the country ^29^ ^,^ ^30^. The selected cities belonged to the Mexican National Program for the Social Prevention of Violence and Crime and were identified as those that required greater short-term attention ^30^. At least one city from each state was chosen, and the sample was representative of the level of each city. The selection of the households was made by probabilistic, stratified, single-stage, and conglomerate sampling. For the final selection of households, three-stage sampling was used (select census tracts within cities, select blocks within census tracts, and select houses within blocks) ^31^.

In the selected households, two questionnaires were applied. The first questionnaire was answered by the household head regardless of their age. It was considered as a household head the person who was part of the household and was recognized as the head by its members, regardless of sex ^30^. To answer the second questionnaire, a family member aged from 12 to 29 years who was not the head of the household was selected. In case of more than one member with these characteristics, only one was randomly chosen. This questionnaire was not applied in households without members with these characteristics. This study found a 13.12% non-response rate, of which 3.49% refer to incomplete interviews, and 9.63%, to households without information despite being inhabited ^32^.

For this study, the population of interest included adolescents and youths. The households included in the sample totaled 80,802. Around half of these households had no adolescents or youths. Participants who answered the questionnaire for adolescents and youths were included in the analysis (n = 40,366). Those who did not answer all questions related to psychological distress (n = 181) and those whose parents interfered with the interview (n = 546) were excluded. The analytical sample consisted of 39,639 adolescents and youths.

### Outcome

The measurement of psychological distress was based on the responses of participants (adolescents and youth) to an 8-item inventory about psychological distress symptoms during the last year that could be answered with “yes” or “no” (Table 1). Exploratory factor analysis (EFA) was performed by the principal component factor method ^33^. Except for one item (“reassuring yourself by hitting objects,” factorial load < 0.4), all were grouped into a single factor (Cronbach’s alpha, α = 0.63). The variable was constructed from the sum of the affirmative answers, obtaining a scale from 0 (no affirmative answers) to 7 (all affirmative answers).

**Table 1:** Factor analysis of psychological distress symptoms among Mexican adolescents and youths aged from 12 to 29 years (n = 39,639).

Items		EFA	Overall sample (%)	Responses according to sex (%)	
				Males (n = 20,260)	Females (n = 19,379)
Eigenvalue		2.27			
Variance (%)		2.27			
Spanish	English				
*En lo que va del año, dime por favor si te ha ocurrido lo siguiente...*	So far this year, please tell me if the following has happened to you...				
1. *Estar inquieto o ansioso*	1. Being restless or anxious	0.60	53.3	50.7	56.2
2. *Tener dificultades para concentrarte o mantener la atención en lo que estás haciendo*	2. Having trouble to concentrate or stay focused on what you are doing	0.58	37.8	35.3	40.4
3. *Tener los músculos tensos o adoloridos por el estrés*	3. Having tense or sore muscles because of stress	0.57	37.4	29.9	45.3
4. *Tener dificultad para dormir o permanecer dormido*	4. Having trouble to sleep or stay asleep	0.60	31.3	28.5	34.2
5. *Sudar con mayor intensidad de lo normal en alguna parte del cuerpo*	5. Sweating more intensely than normal in any part of the body	0.43	13.8	15.8	11.8
6. *Tranquilizarte golpeando objetos*	6. Reassuring yourself by hitting objects	0.39	8.1	9.9	6.3
7. *Estar muy enfermo*	7. Being very sick	0.41	10.6	9.0	12.3
8. *Estar muy triste o deprimido, o sentirte muy solo*	8. Being very sad or depressed or feeling very lonely	0.62	19.3	13.8	25.0

%: weighted estimates; EFA: exploratory factor analysis.

Note: bold values are the factorial weights of the items with values of 0.40 or more.

### Exposure

The secondary sample (according to household heads) was used to measure violent community environments. This community sample managed to adjust contextual variables to assess the exposure. These variables were the only ones that were generated using information from household heads rather than from the participants. In this questionnaire, a scale on 16 acts of incivilities and crimes in the neighborhood was included ^34^. It had four response options: never (scored with 0), infrequent, frequent, and very frequent (scored with 3). Data from the household heads who completed the scale was used regardless of whether adolescents or youths lived in the household and regardless of the age of the household head (n = 80,802). The EFA was conducted (principal component factor method with oblique rotation) and three factors emerged (Table 2). Then, three exposure variables were constructed: (a) social disorder, with eight items (e.g., making noise, drinking alcohol on the streets, α = 0.77); (b) vandalism, with three items (e.g., tagging walls, scratching cars, α = 0.57); and (c) criminality, with four items (e.g., robbing or assaulting and threatening or extorting, α = 0.75).

**Table 2:** Factor analysis of living in a disordered and violent community environments reported by household heads in Mexico (n = 80,802).

Items		EFA			Distribution by responses (%)			
		1	2	3	Never	Infrequent	Frequent	Very frequent
Eigenvalue		4.21	3.32	2.85				
Variance (%)		26.33	20.73	17.80				
Spanish	English							
*En lo que va del año, ¿qué tan frecuente ha observado gente en su colonia o bairro...*	So far this year, how often have you observed people in your neighborhood...							
1. *Haciendo ruido?*	1. Making noise?	0.54	-0.29	0.28	30.7	34.5	18.2	16.6
2. *Grafiteando paredes o rayando autos?*	2. Tagging walls or scratching cars?	0.00	0.14	0.69	77.0	12.2	6.8	4.0
3. *Rompiendo ventanas de casas, negocios, autos u otros objetos?*	3. Breaking windows of houses, businesses, cars, or other objects?	-0.12	0.18	0.77	87.6	7.8	3.3	1.3
4. *Jugando arrancones?*	4. Participating in street races?	0.04	-0.00	0.59	88.3	6.9	3.4	1.5
5. *Tomando alcohol en la calle?*	5. Drinking alcohol on the street?	0.71	-0.13	0.12	49.4	20.5	17.9	12.2
6. *Vendiendo productos pirata?*	6. Selling counterfeit products?	0.56	0.08	-0.04	78.0	11.0	7.2	3.8
7. *Vendiendo drogas?*	7. Selling drugs?	0.68	0.21	-0.17	85.1	5.7	5.2	4.0
8. *Consumiendo drogas?*	8. Using drugs?	0.77	0.13	-0.13	69.3	12.2	10.6	8.0
9. *Bloqueando la calle?*	9. Blocking the street?	0.46	-0.01	0.11	86.8	7.9	3.2	2.0
10. *Peleando entre pandillas?*	10. Gang fighting?	0.42	0.08	0.24	83.5	9.7	4.2	2.7
11. *Discutiendo o peleando entre vecinos?*	11. Arguing or fighting between neighbors?	0.54	-0.02	0.08	78.4	16.3	3.7	1.5
12. *Prostituyéndose?*	12. Working as a prostitute?	0.33	0.20	-0.09	95.7	2.4	1.1	0.7
13. *Asaltando o robando casas, negocios o vehículos?*	13. Robbing or assaulting homes, businesses, or vehicles?	-0.07	0.76	0.18	71.5	15.3	9.1	4.1
14. *Asaltando o robando a personas en la calle?*	14. Assaulting or robbing people on the street?	-0.01	0.80	0.09	72.6	13.7	9.3	4.5
15. *Amenazando o extorsionando?*	15. Threatening or extorting?	0.05	0.67	0.04	90.4	5.4	2.9	1.3
16. *Disparando algún tipo de arma de fuego?*	16. Firing a firearm?	0.23	0.54	-0.04	86.2	8.6	3.5	1.7

%: weighted estimates; EFA: exploratory factor analysis.

Note: bold values are the factorial weights of the items with values of 0.40 or more.

The contextual variables were constructed at the level of census tracts. In Mexico, the census tracts are called “basic geostatistical area” (AGEB, acronym in Spanish). AGEB includes from 1 to 50 blocks in which the population is socioeconomically homogeneous ^35^. The homogeneity of the responses within the AGEB was evaluated via intraclass correlation coefficient (ICC). For social disorder, it totaled 0.28 (95% confidence interval - 95%CI: 0.27, 0.29), for vandalism, 0.16 (95%CI: 0.15, 0.16), and for criminality, 0.30 (95%CI: 0.29, 0.31). The values of household heads’ responses for every variable were aggregated to obtain a value for each census tract. Subsequently, for each variable, the median by AGEB was estimated and was assigned to each participant according to the AGEB in which they lived. Social disorder was categorized in quartiles. For vandalism and criminality, three categories were formed according to the assigned values: 0, more than 0 to 1, and more than 1.

### Modifying variables

It was considered that sex and age could be modifying variables between living in a disordered and violent community (social disorder, vandalism, and criminality) and psychological distress. When the modifying effect was identified, the main analysis was performed stratifying by sex, age, or both. Otherwise, sex and age were included in the analysis as confounding variables. For sex, it was considered male and female and for age, participants were separated into those who were aged from 12 to 18 years (adolescents) and those from 18 to 29 years (youths).

### Interpersonal violence

Interpersonal community violence identifies victims of any type of community violence during the last year. It was constructed with the responses to the question: “During 2014 (from January up to now), did any of the following situations happen to you...?”, which included 10 situations: teasing, hiding personal objects, physical aggression, robbery without violence, robbery with violence, threats, extortion, virtual violence, or sexual abuse (touching and sexual encounters). The situations were considered when participants experienced them in public places such as squares, markets, parks, or on the street.

### Covariates

Occupation was distributed in four categories (study, work, study and work, and neither study nor work). Depending on whether participants had moved to the neighborhood, four categories were formed: always lived in the area, moved more than 10 years ago, moved from 5 to 10 years ago, or moved less than 5 years ago.

Economic hardships were measured according to answers to the 10-item scale, in which one item had insufficient answers and was excluded (Table 3). The response options for the other nine items were: true, false, does not apply, and does not know or did not answer. True or false answers were used. In total, seven items were selected because they belong to the same factor in the EFA with oblique rotation (e.g., have enough food, have enough money to buy clothes and shoes). The number of hardships was added and three groups were formed (none, 1 or 2, and 3 to 7 hardships). Household heads’ education was classified into four categories: primary or less (including primary and no instruction), junior high school (including technical career with completed junior high school), high school (including normal basic, high school, and high school with completed technical career), and bachelor or more (including master’s and PhD degrees). Overall, four geographic regions were defined according to the ubication of the cities (north, west, center, and south).

**Table 3:** Factor analysis of economic hardships Mexican adolescents and youth aged from 12 to 29 years (n = 39,953).

Items		EFA		Affirmative answers (%)
		1	2	
Eigenvalue		3.16	1.04	
Variance (%)		3.13	1.17	
Spanish	English			
*De las situaciones mencionadas, te pido por favor que me digas si las consideras ciertas o falsas. ¿En tu casa...*	Of the situations mentioned, I ask you to please tell me if you consider them true or false. In your house...			
1. *Tienen suficiente comida para todos, todos los días?*	1. Have enough food for everyone, every day?	0.49	-0.12	95.2
2. *Tienen alguna deuda (con el banco, casas de préstamo, parientes, amigos o vecinos)?*	2. Have any debt (with the bank, loan houses, relatives, friends, or neighbors)?	-0.00	0.70	35.1
3. *Tienen dinero suficiente para comprar ropa y calzado?*	3. Have enough money to buy clothes, and shoes?	0.73	0.05	81.3
4. *Para cubrir sus necesidades tienen que trabajar los siete días de la semana?*	4. Have to work seven days a week to cover your needs?	0.05	0.69	55.6
5. *Tienen dinero suficiente para divertirse o convivir?*	5. Have enough money to have fun or hang out?	0.72	0.14	74.0
6. *Pueden pagar las medicinas y atención médica que requieren?*	6. Can pay for the medicines and medical attention required?	0.69	0.18	90.1
7. *Tienen dinero suficiente para pagar tus necesidades escolares (útiles, uniformes, cuotas, etc)?*	7. Have enough money to pay for your school needs (supplies, uniforms, fees, etc)?	0.72	0.12	87.6
8. *Les alcanza para darse sus gustos?*	8. Is it enough for them to indulge themselves?	0.65	0.20	68.7
9. *Si la vivienda es propia, tienen suficiente dinero para darle mantenimiento a la casa?*	9. If is your own house, have enough money to maintain the house?	0.62	0.08	75.6

%: weighted estimates; EFA: exploratory factor analysis.

Note: bold values are the factorial weights of the items with values of 0.40 or more.

After the interviews, interviewers indicated the level of intervention from guardians. An ordinal variable was generated with a scale from 0 (in which the adult was absent) to 5 (in which the adult was interested in knowing the questionnaire without interfering with the interviews).

### Statistical analysis

Analysis was run on Stata, version 15 (https://www.stata.com). Considering the survey design, weighted relative frequencies were estimated to know the distribution of each variable. Weighted means of distress symptoms were obtained according to exposures.

To select the variables to adjust the models, means of psychological distress were estimated according to each covariate (data not shown). Comparisons between categories were made using 95%CI. Moreover, weighted relative frequencies of the community violence variables were estimated according to covariates (data not shown). The chi-squared test was used to identify differences between categories. To identify the relationship between exposure to violent community environments and psychological distress symptoms, a first crude model was carried out. A second model was adjusted for sex, age, occupation, economic hardships, region, moving from the neighborhood, and parental intervention. The third model was adjusted for the variables of the second model plus interpersonal community violence. Moreover, 95%CI were estimated for each model.

Multilevel linear regression models were estimated using the identification of census tracts as the random intercept. These models recognize and consider the homogeneity of participants within the AGEB since living in the same AGEB share the exposure to disorder, community violence, and other social characteristics ^36^. For psychological distress, an ICC of 0.07 (95%CI: 0.06, 0.08) was obtained, which justifies the use of multilevel models considering the AGEB as a random intercept.

To test whether sex and age were modifying variables, the same models were estimated including the interactions of these variables with the three exposure variables. An interaction was considered significant if p < 0.100. This cutoff for significance level was selected to identify small effects on interactions ^37^. Moreover, confidence intervals were estimated to know the certainty of the identified interactions ^38^. If the interactions were significant, analyses were carried out that were stratified by sex, age, or both.

### Ethical aspects

Although participation in the ECOPRED was compulsory because it was part of the governmental system of information ^29^, no penalty in case was applied for not participating. Thus, 100% of the responses were unable to be obtained ^32^. Confidentiality was guaranteed to participants. The ethics of the conducted analysis was granted by the Ethics Committee at the Division of Biological and Health Sciences at the Metropolitan Autonomous University Xochimilco (Mexico).

## Results

The mean age of participants was 18.7 years (standard deviation - SD: 4.7), of which just over half were adolescents. The proportion of males was slightly higher than that of females (51.1% vs. 48.9%). Around half of participants were studying and reported no economic hardships. Most household heads have secondary education or less. Just over 40% had always lived in the same neighborhood, whereas about a fifth had changed neighborhood 10 or more years ago (Table 4). The highest proportion came from northern Mexico. Just over 20% had experienced some type of interpersonal violence in the community. More than half of the interviews were conducted without the presence of guardians.

**Table 4:** Sociodemographic characteristics of Mexican adolescents and youths aged from 12 to 29 years according to sex and age.

Characteristics	Overall sample (%) (n = 39,639)	Differences by sex (%)		Males (%)		Females (%)	
		Males (n = 20,260)	Females (n = 19,379)	Adolescents (n = 10,634)	Youths (n = 9,626)	Adolescents (n = 10,374)	Youths (n = 9,005)
Age (years)							
12-15	28.7	27.4	30.1	54.5	-	57.7	-
16-18	22.5	22.9	22.1	45.5	-	42.3	-
19-23	29.9	30.3	29.3	-	61.0	-	61.4
24-29	18.9	19.4	18.4	-	39.0	-	38.6
Occupation							
Study	51.1	47.9	54.6	74.8	20.7	82.3	24.4
Work	27.7	32.7	22.5	10.3	55.3	4.5	42.1
Study and work	10.4	12.8	7.9	8.8	16.8	4.0	12.2
Neither studies nor works	10.7	6.7	15.0	6.2	7.2	9.2	21.4
Moving from the neighborhood (years)							
Never	43.7	42.8	44.7	46.0	39.6	47.8	41.3
> 10	19.4	20.7	18.1	12.4	29.0	11.8	25.0
5-10	19.1	19.3	18.9	23.5	15.2	22.1	15.4
< 5	17.7	17.2	18.3	18.1	16.2	18.3	18.3
Economic hardships							
None	51.1	52.2	50.0	51.0	53.3	50.8	49.0
1 or 2	28.4	29.1	27.8	30.7	27.4	28.0	27.5
3 to 7	20.5	18.8	22.2	18.3	19.2	21.2	23.5
Household head education							
Primary or less	29.6	30.2	29.0	27.8	32.7	27.1	31.0
Junior high school	32.4	32.6	32.2	34.4	30.6	35.8	28.2
High school	18.6	18.1	19.1	20.5	15.8	19.6	18.6
Bachelor or more	19.4	19.1	19.7	17.2	20.9	17.4	22.1
Region							
North	36.9	36.7	37.1	38.3	35.1	38.5	35.5
West	26.7	26.9	26.6	26.5	27.3	26.3	26.9
Center	24.4	24.4	24.4	22.8	26.0	23.4	25.4
South	12.0	11.9	12.0	12.4	11.5	11.8	12.2
Parental intervention							
Was not present	62.3	63.3	61.3	49.9	76.7	49.9	73.7
Was present in another room	19.5	19.8	19.2	24.3	15.2	22.0	16.2
Was present without paying visual attention	10.5	10.1	10.8	14.9	5.3	15.0	6.2
Was present and only paying visual attention	6.5	5.6	7.4	8.7	2.4	11.1	3.3
Was present and sought to know the instrument	1.2	1.2	1.3	2.1	0.4	2.0	0.5
Interpersonal community violence							
No	77.5	76.1	79.0	79.4	72.8	81.7	76.1
Yes	22.5	23.9	21.0	20.6	27.2	18.3	23.9

%: weighted estimates.

Note: bold values represent significant differences when comparing males with females and adolescents with youths. Significance level: 0.05 (p < 0.05).

Compared to males, more females studied or neither studied nor worked, had more economic hardships, and experienced less community interpersonal violence. More adolescents were studying, and, compared to them, youths have been victims of interpersonal violence in a higher proportion.

The average of psychological distress symptoms in females and youths exceeded that of males and adolescents, respectively (Table 5). Psychological distress symptoms tended to be higher in the fourth quartile of social disorder than in the first quartile but were significant only in adolescent females. Among males in the category with higher vandalism showed more psychological distress symptoms. Adolescent females showed the highest mean of distress symptoms if vandalism was higher, whereas, female youths, showed the opposite. In adolescents, psychological distress was higher when more criminal acts occurred. Youths had more psychological distress symptoms when fewer criminal acts occurred. Adolescents and youths of both sexes who experienced interpersonal community violence had greater psychological distress than those who reported no victimization from this type of act.

**Table 5:** Psychological distress according to living in disordered and violent community environments and interpersonal community violence in Mexican adolescents and youths aged from 12 to 29 years according to sex and age.

	Overall sample (x̅)		Males (x̅)		Females (x̅)	
	Males	Females	Adolescents	Youths	Adolescents	Youths
Overall	1.83	2.25	1.75	1.91	2.20	2.31
Social disorder (quartiles)						
I	1.82	2.15 *	1.73	1.91	2.09 *	2.22
II	1.79	2.25	1.69	1.90	2.21	2.30
III	1.83	2.16 **	1.81	1.84	2.07 **	2.25
IV	1.87	2.38	1.79	1.97	2.34	2.42
Vandalism						
0	1.80	2.26	1.70	1.90	2.21	2.32
0-1	1.87	2.21	1.85	1.88	2.12	2.30
> 1	1.94	2.25	1.84	2.04	2.25	2.26
Criminality						
0	1.83	2.28	1.74	1.92	2.20	2.37
0-1	1.84	2.19	1.73	1.94	2.14	2.24
> 1	1.83	2.22	1.79	1.88	2.24	2.20
Interpersonal community violence						
No	1.70	2.06	1.62	1.79	2.02	2.12
Yes	2.25 ***	2.96 ***	2.27 ***	2.24 ***	3.03 ***	2.90 ***

X̅: weighted average.

* Difference when comparing quartile I versus quartile IV considering their 95% confidence intervals;

** Difference when comparing quartile III versus quartile IV considering their 95% confidence intervals;

*** Differences when comparing yes versus no interpersonal community violence.

Interactions with sex occurred for social disorder, vandalism, and criminality, whereas, with age, the interaction occurred only with criminality in females (Table 6).

**Table 6:** Assessment of interactions between exposure with sex and age.

	β	95%CI	p-value
Overall sample			
Social disorder (quartiles) * sex			
II*female	0.056	-0.027, 0.138	0.187
II*female	0.050	-0.057, 0.156	0.363
IV*female	0.077	-0.012, 0.166	0.091
Vandalism * sex			
0-1*female	-0.021	-0.111, 0.068	0.640
> 1*female	-0.144	-0.256, -0.032	0.011
Criminality * sex			
0-1*female	-0.118	-0.209, -0.027	0.011
> 1*female	-0.085	-0.167, -0.002	0.044
Males			
Social disorder (quartiles) * sex			
II*female	0.016	-0.096, 0.129	0.778
II*female	-0.114	-0.259, 0.030	0.120
IV*female	0.001	-0.119, 0.122	0.984
Vandalism * sex			
0-1*female	-0.060	-0.180, 0.060	0.328
> 1*female	0.066	-0.085, 0.218	0.388
Criminality * sex			
0-1*female	0.005	-0.117, 0.128	0.931
> 1*female	-0.093	-0.204, 0.019	0.104
Females			
Social disorder (quartiles) * sex			
II*female	0.068	-0.055, 0.192	0.277
II*female	0.091	-0.068, 0.250	0.264
IV*female	-0.020	-0.154, 0.113	0.765
Vandalism * sex			
0-1*female	-0.064	-0.198, 0.069	0.346
> 1*female	0.053	-0.113, 0.220	0.529
Criminality * sex			
0-1*female	-0.136	-0.272, 0.001	0.051
> 1*female	-0.117	-0.240, 0.006	0.061

95%CI: 95% confidence interval; β: regression coefficient.

Note: all models were adjusted for youth occupation, economic hardships, region, moving from the neighborhood, and parental intervention.

Table 7 shows multilevel models. Participants had higher psychological distress in the highest quartiles of social disorder; these differences remained after adjusting for other covariables in both sexes, except in males when adjusting for interpersonal community violence. The men under a higher occurrence of acts of vandalism had higher psychological distress symptoms than their counterparts. This difference remained after adjustment for all variables. Conversely, men who lived in places with more criminality had less psychological distress symptoms than those who lived in places with low criminality, but it emerged only after adjusting for all covariates. Female youths in an area with higher criminality were associated with fewer psychological distress symptoms in both unadjusted and adjusted models.

**Table 7:** Multilevel models considering symptoms of psychological distress as a dependent variable and living in a disordered and violent community environment variables as independent variables.

	Model 0		Model 1		Model 2	
	β	95%CI	β	95%CI	β	95%CI
Social disorder						
Males (quartiles)						
II	0.07	0.01, 0.12	0.06	0.00, 0.12	0.05	-0.01, 0.10
III	0.09	0.01, 0.16	0.08	0.01, 0.16	0.05	-0.02, 0.13
IV	0.09	0.02, 0.15	0.08	0.01, 0.14	0.03	-0.03, 0.10
Females (quartiles)						
II	0.14	0.07, 0.20	0.12	0.05, 0.18	0.10	0.04, 0.17
III	0.15	0.06, 0.23	0.12	0.04, 0.21	0.10	0.02, 0.18
IV	0.17	0.10, 0.24	0.16	0.09, 0.24	0.12	0.05, 0.19
Vandalism						
Males						
0-1	0.01	-0.05, 0.08	0.01	-0.05, 0.07	-0.01	-0.07, 0.06
> 1	0.12	0.04, 0.20	0.12	0.04, 0.20	0.09	0.01, 0.17
Females						
0-1	-0.00	-0.07, 0.07	-0.00	-0.07, 0.07	-0.03	-0.10, 0.04
> 1	-0.01	-0.10, 0.07	-0.01	-0.10, 0.08	-0.03	-0.12, 0.05
Criminality						
Males						
0-1	0.03	-0.03, 0.10	0.02	-0.04, 0.09	0.01	-0.06, 0.07
> 1	-0.02	-0.08, 0.04	-0.04	-0.10, 0.03	-0.07	-0.13, -0.01
Females						
Adolescents						
0-1	-0.03	-0.12, 0.07	-0.03	-0.12, 0.07	-0.04	-0.13, 0.05
> 1	-0.06	-0.14, 0.03	-0.04	-0.13, 0.05	-0.06	-0.15, 0.03
Youths						
0-1	-0.16	-0.27, -0.06	-0.15	-0.26, -0.05	-0.19	-0.29, -0.09
> 1	-0.18	-0.27, -0.08	-0.16	-0.25, -0.06	-0.23	-0.33, -0.13

95%CI: 95% confidence interval; β: regression coefficient.

Note: bold values represent significant differences. Significance level: 0.05 (p < 0.05).

Reference groups: quartile 1 (social disorder) or 0 (vandalism and criminality). Model 0 without adjustment for other variables. Model 1 adjusted for youth occupation, economic hardships, region, moving from the neighborhood, and parental intervention. Model 2 adjusted for the variables included in the model 1 plus interpersonal community violence.

## Discussion and conclusion

A representative sample of Mexican adolescents and youths living in different cities of the country showed a relation between exposure to three patterns of disordered and violent community environments and symptoms of psychological distress. These patterns refer to social disorder, vandalism, and criminality, which, although they may be related, should be studied separately since the association of each one with negative mental health outcomes may be different. Based on the existing evidence ^13^ ^,^ ^14^, it was expected that the participants most exposed to disordered and violent communities would also have more psychological distress. Our study found this relationship between social disorder and psychological distress in both sexes and between vandalism and psychological distress in men. Criminality was unrelated to psychological distress or showed an opposite relationship. It was possible to recognize age and sex as modifiers of the relationship between disordered and violent community environments and psychological distress.

The relationship between social disorder and vandalism with psychological distress was independent of interpersonal experiences of community violence (except for social disorder in males). These acts can produce discomfort, increase fear, and generate a threat perception even without being a victim of interpersonal violence, which would favor the development of psychological distress ^10^.

Sex modified the relationship between social disorder and vandalism with psychological distress. Although males and females showed that psychological distress was higher in those who lived with higher social disorder, the relationship disappeared in males after adjusting for interpersonal community violence. Regarding vandalism, only men who lived in communities in which these acts occurred more frequently had more psychological distress. Perhaps, these differences could be explained by men’s greater exposure to this disorder in community environments because they spend more time on the streets and participate in disordered and vandalistic acts more frequently than women ^27^. Thus, men may be used to social disorder, which would only increase psychological distress if accompanied by interpersonal community violence. However, vandalism increases psychological distress regardless of the interpersonal community violence. Women tend to be afraid and perceive insecurity in the streets ^39^, which makes them take some precautions and prefer to stay at home or avoid public places ^40^. But even with these precautions, social disorder cannot be avoided, which causes discomfort and increases psychological distress.

Contrary to our expectations, criminality was not related to psychological distress. Even youths showed an inverse relationship. These results agree with what was observed in youths in the United States ^23^ ^,^ ^24^ ^,^ ^25^, in which places with greater community violence had fewer effects on mental health. This inverse association has potential explanations. First, at the contextual level, it has been observed that communities that have experienced traumatic events tend to increase their social cohesion. Closer relationships within a community could contribute to overcoming adverse situations ^41^. A similar phenomenon could occur in communities with criminal acts ^25^, i.e., this type of adversity generates greater social cohesion, which benefits inhabitants by enabling them to carry out their activities without suffering the effects of criminality.

Second, at the individual level, it is likely that the people who stay in this type of environment have undergone adaptation or normalization ^42^. In some populations, exposure to violence could generate biological hypo-reactivity, reducing bodily responses to this type of event ^43^. Most ECOPRED participants had never changed neighborhoods or had moved 10 years ago or more, which would explain that they could have experienced a process of adaptation and normalized the crimes occurring in their community. Moreover, criminality tends to remain stable in neighborhoods ^44^, which favors the adaptation process.

Third, it is possible that people have changed their behavior, enabling them to live with greater tranquility. Echoing the theory of protection motivation ^45^, individuals who are known to be vulnerable and are aware of the risk to which they are exposed by living in an environment in which criminal acts occur probably take the necessary measures in such a way that, although they live in an environment with exposure to violence, they can experience it as safe. Finally, they perceive the probability of being victim of this type of act as low. The frequency of criminal acts was lower than the acts of vandalism and social disorder. Therefore, and considering the changes in inhabitants’ behavior, the chances of being a victim of criminal acts decrease, reducing psychological distress.

Our study has the following strengths: (1) a representative sample of household heads, which enabled the construction of contextual variables on disordered and violent community environments; (2) the investigation of different dimensions of disordered and violent community environments were investigated enabled us to recognize specific forms of exposure to disordered and violent communities that could affect mental health outcomes in different ways; (3) the analysis stratified by sex and age helped to evaluate the effect of these variables on the associations under examination; and (4) the development of this study represented 47 Mexican cities, which included at least one city in each Federal Entity.

However, some important limitations are linked to the secondary analysis of the data: (1) the inventory of psychological distress used still requires validation. Most symptoms in this inventory were associated with anxiety, and only one was related to depression. However, although an unvalidated scale, its items resemble those of other scales used to measure psychological distress (e.g., *Kessler 6*) ^46^; (2) because of its cross-sectional design, we were unable to establish any causal relationships. Although psychological distress would hardly explain the indices of community violence, people who suffered from a violent environment may have moved elsewhere; and (3) notably, it is not possible to distinguish whether some participants were the perpetrators of the studied acts of community violence with the available information.

In summary, Mexican urban youths showed a relationship between living in disordered and violent community environments with psychological distress over and above individual victimization experiences. Females from environments in which acts of social disorder occur and males from communities with higher levels of vandalism had higher psychological distress. Criminality was unrelated or had an inverse relationship with psychological distress, particularly in women.

These findings are important since they provide the necessary evidence to address the disorder and violence experienced in communities. It is necessary to generate strategies that reduce social disorder and vandalism within communities, which will favor a decrease in psychological distress in adolescents and youths. Moreover, these results allow us to propose future research to further develop our knowledge of the relationship between living in a disordered and violent community environment and its effects on mental health. It would be important to use previously validated instruments to assess psychological distress and other problems related to mental health to evaluate consistency with our results. Moreover, it would be necessary to compare what happens between young people who participate as perpetrators of acts of community violence and those who avoid participating in these acts. Finally, it is important to consider the social factors that could influence this relationship; it is necessary to evaluate the role of variables such as social capital or social cohesion as possible moderators of the identified relationships.

## References

[1] Organización Panamericana de la Salud (2018). La carga de los trastornos mentales en la Región de las Américas, 2018.

[2] Instituto Nacional de Estadística y Geografía (2017). Encuesta Nacional de los Hogares 2017.

[3] Instituto Nacional de Estadística y Geografía (2018). Encuesta Nacional de Victimización y Percepción sobre Seguridad Pública (ENVIPE) 2018.

[4] Instituto Nacional de Estadística y Geografía (2022). Encuesta Nacional de Victimización y Percepción sobre Seguridad Pública (ENVIPE) 2022.

[5] Aisenberg E, Herrenkohl T (2008). Community violence in context risk and resilience in children and families. J Interpers Violence.

[6] Instituto Nacional de Estadística y Geografía (2017). Encuesta Nacional de Victimización y Percepción sobre Seguridad Pública (ENVIPE) 2017.

[7] Frías S, Finkelhor D (2017). Victimizations of Mexican youth (12-17 years old) a 2014 national survey. Child Abuse Negl.

[8] Aisenberg E, Trickett P, Mennen F, Saltzman W, Zayas L (2007). Maternal depression and adolescent behavior problems an examination of mediation among immigrant Latino mothers and their adolescent children exposed to community violence. J Interpers Violence.

[9] Cooley-Quille MR, Turner SM, Beidel DC (1995). The emotional impact of children's exposure to community violence a preliminary study. J Am Acad Child Adolesc Psychiatry.

[10] O'Brien DT, Farrell C, Welsh BC (2018). Broken (windows) theory: a meta-analysis of the evidence for the pathways from neighborhood disorder to resident health outcomes and behaviors.. Soc Sci Med.

[11] Hedayati Marzbali M, Safizadeh M, Tilaki MJM, Abdullah A (2021). Does facilitating human-place bonds alleviate the negative effects of incivilities on health. Sustainability.

[12] Foster H, Brooks-Gunn J (2009). Toward a stress process model of children's exposure to physical family and community violence. Clin Child Fam Psychol Rev.

[13] Fowler P, Tompsett C, Braciszewski J, Jaques-Tiura A, Baltes B (2009). Community violence a meta-analysis on the effect of exposure and mental health outcomes of children and adolescents. Dev Psychopathol.

[14] McDonald C, Richmond T (2008). The relationship between community violence exposure and mental health symptoms in urban adolescents. J Psychiatr Ment Health Nurs.

[15] Cuartas J, Roy A (2019). The latent threat of community violence indirect exposure to local homicides and adolescents' mental health in Colombia. Am J Community Psychol.

[16] Baranyi G, Cherrie M, Curtis S, Dibben C, Pearce J (2020). Changing levels of local crime and mental health a natural experiment using self-reported and service use data in Scotland. J Epidemiol Community Health.

[17] Cooley-Strickland M, Quille T, Griffin R, Stuart E, Bradshaw C, Furr-Holden D (2011). Efectos de la exposición de los adolescentes a la violencia en la comunidad el Proyecto MORE. Psychosocial Intervention.

[18] Cohen S (2004). Social relationships and health. Am Psychol.

[19] Miliauskas CR, Faus DP, Cruz VL, Vallaperde JGRN, Junger W, Lopes CS (2022). Community violence and internalizing mental health symptoms in adolescents a systematic review. BMC Psychiatry.

[20] Grinshteyn E, Xu H, Manteuffel B, Ettner S (2018). The associations of area-level violent crime rates and self-reported violent crime exposure with adolescent behavioral health. Community Ment Health J.

[21] Dupéré V, Leventhal T, Vitaro F (2012). Neighborhood processes, self-efficacy, and adolescent mental health. J Health Soc Behav.

[22] McKelvey LM, Whiteside-Mansell L, Bradle RH, Casey PH, Conners-Burrow NA, Barret KW (2011). Growing up in violent communities do family conflict and gender moderate impacts on adolescents'psychosocial development?. J Abnorm Child Psychol.

[23] Goldman-Mellor S, Margerison-Zilko C, Allen K, Cerda M (2016). Perceived and objectively-measured neighborhood violence and adolescent psychological distress. J Urban Health.

[24] Boxer P, Sloan-Power E, Piza E, Schappell A (2014). Using police data to measure children's exposure to neighborhood violence a new method for evaluating relations between exposure and mental health. Violence Vict.

[25] Colson K, Galin J, Ahern J (2016). Spatial proximity to incidents of community violence is associated with fewer suicides in urban California. J Urban Health.

[26] González S La salud mental en México. Director General de los Servicios de Salud Mental..

[27] González-Pérez G, Vega-López M (2019). Homicidio juvenil y su impacto en la esperanza de vida masculina variaciones geográficas y factores asociados. Salud Colect.

[28] Comisión Nacional de los Derechos Humanos (2018). Informe especial adolescentes: vulnerabilidad y violencia.

[29] Instituto Nacional de Estadística y Geografía (2015). Encuesta de Cohesión Social para la Prevención de la Violencia y la Delincuencia 2014: síntesis metodológica.

[30] Instituto Nacional de Estadística y Geografía (2015). Encuesta de Cohesión Social para la Prevención de la Violencia y la Delincuencia 2014: marco conceptual.

[31] Instituto Nacional de Estadística y Geografía (2015). Encuesta de Cohesión Social para la Prevención de la Violencia y la Delincuencia 2014: diseño muestral.

[32] Instituto Nacional de Estadística y Geografía (2015). Encuesta de Cohesión Social para la Prevención de la Violencia y la Delincuencia 2014: informe operativo.

[33] Acock A (2013). Discovering structural equation modeling using Stata.

[34] Shin S, McDonalda S, Conley D (2018). Patterns of adverse childhood experiences and substance use among young adults a latent class analysis. Addict Behav.

[35] Instituto Nacional de Estadística y Geografía (2016). Manual de cartografía geoestadística.

[36] Fitzmaurice G, Laird N, Ware J (2011). Applied longitudinal analysis.

[37] Durand CP (2013). Does raising type 1 error rate improve power to detect interactions in linear regression models A simulation study. PLoS One.

[38] Amrhein V, Greenland S (2022). Discuss practical importance of results based on interval estimates and p-value functions, not only on point estimates and null p-values. Journal of Information Technology.

[39] Caridade S, Magalhães M, Azevedo V, Dinis MAP, Maia RL, Estrada R (2022). Predicting frequent and feared crime typologies individual and social/environmental variables, and incivilities. Soc Sci (Basel).

[40] Soto-Villagrán P (2012). El miedo de las mujeres a la violencia en la ciudad de México Una cuestión de justicia espacial. Revista INVI.

[41] Sweet S (1998). The effect of a natural disaster on social cohesion a longitudinal study. Int J Mass Emerg Disasters.

[42] Gaylord-Harden N, Cunningham J, Zelencik B (2011). Effects of exposure to community violence on internalizing symptoms does desensitization to violence occur in African American youth?. J Abnorm Child Psychol.

[43] Cerda-Molina A, Borráz-León J, Mayagoitia-Novales L, Gaspar Del Río A (2017). Reactividad del cortisol y salud mental en adultos expuestos a violencia temprana revisión sistemática. Rev Panam Salud Pública.

[44] Weisburd D, Bushway S, Lum C, Yang S (2004). Trajectories of crime at places a longitudinal study of street segments in the city of Seattle. Criminology.

[45] Rogers R, Cacioppo JT, Perry RE (1983). Social psychophysiology: a source book.

[46] Kessler R, Andrews G, Colpe L, Hiripi E, Mroczek D, Normand S (2002). Short screening scales to monitor population prevalences and trends in non-specific psychological distress. Psychol Med.

